# A review of simulation models for the long-term management of type 2 diabetes in low-and-middle income countries

**DOI:** 10.1186/s12913-021-07324-0

**Published:** 2021-12-06

**Authors:** Elton Mukonda, Susan Cleary, Maia Lesosky

**Affiliations:** 1 Division of Epidemiology and Biostatistics, School of Public Health and Family Medicine, Faculty of Health Sciences, University of Cape Town, Observatory, Cape Town, 7925 South Africa; 2 Health Economics Unit, School of Public Health & Family Medicine, University of Cape Town, Cape Town, South Africa

**Keywords:** Type-2 diabetes, Economic evaluation, Simulation models, Low-and-middle income countries

## Abstract

**Introduction:**

The burden of type 2 diabetes is steadily increasing in low-and-middle-income countries, thereby posing a major threat from both a treatment, and funding standpoint. Although simulation modelling is generally relied upon for evaluating long-term costs and consequences associated with diabetes interventions, no recent article has reviewed the characteristics and capabilities of available models used in low-and-middle-income countries. We review the use of computer simulation modelling for the management of type 2 diabetes in low-and-middle-income countries.

**Methods:**

A search for studies reporting computer simulation models of the natural history of individuals with type 2 diabetes and/or decision models to evaluate the impact of treatment strategies on these populations was conducted in PubMed. Data were extracted following Preferred Reporting Items for Systematic Reviews and Meta-Analyses (PRISMA) guidelines and assessed using modelling checklists. Publications before the year 2000, from high-income countries, studies involving animals and analyses that did not use mathematical simulations were excluded. The full text of eligible articles was sourced and information about the intervention and population being modelled, type of modelling approach and the model structure was extracted.

**Results:**

Of the 79 articles suitable for full text review, 44 studies met the inclusion criteria. All were cost-effectiveness/utility studies with the majority being from the East Asia and Pacific region (*n* = 29). Of the included studies, 34 (77.3%) evaluated the cost-effectiveness of pharmacological interventions and approximately 75% of all included studies used HbA1c as one of the treatment effects of the intervention. 32 (73%) of the publications were microsimulation models, and 29 (66%) were state-transition models. Most of the studies utilised annual cycles (*n* = 29, 71%), and accounted for costs and outcomes over 20 years or more (*n* = 38, 86.4%).

**Conclusions:**

While the use of simulation modelling in the management of type 2 diabetes has been steadily increasing in low-and-middle-income countries, there is an urgent need to invest in evaluating therapeutic and policy interventions related to type 2 diabetes in low-and-middle-income countries through simulation modelling, especially with local research data. Moreover, it is important to improve transparency and credibility in the reporting of input data underlying model-based economic analyses, and studies.

**Supplementary Information:**

The online version contains supplementary material available at 10.1186/s12913-021-07324-0.

## Introduction

Type-2 Diabetes (T2D) has a rapidly increasing burden across low-and-middle-income countries (LMICs) and is therefore a major healthcare concern from both a treatment and a funding perspective [1]. The International Diabetes Federation (IDF) [2] indicates that diabetes affected 463 million people worldwide in 2019, of which about 90% was attributable to T2D, and up to 80% of people living with diabetes come from LMICs [2, 3]. The IDF also estimates that the number of people living with diabetes will grow to 700 million by 2045, with the greatest increase in prevalence expected to occur in Africa, the Middle East, South-East Asia and Central America [2]. Diabetes-related mortality accounted for up to 11% of all-cause mortality among people aged 20-79 in 2019 [2], while the 2017 Global Burden of Disease study identified diabetes as the fourth leading cause of disability [4].

In addition to morbidity and mortality, diabetes causes substantial economic burden for individuals, society, and health systems [5–7]. In 2019, diabetes-related health expenditure was estimated to be US$760 billion [2]. While high income countries (HICs) accounted for a large proportion of this amount (90%), the significant increases in disease prevalence expected in LMICs mean they will likely carry a large proportion of future global diabetes health expenditure [2, 4]. Given that healthcare systems and the available (limited) resources are, for the most part, directed towards communicable disease management in LMICs [8], it is imperative that effective and cost-effective interventions are identified to reduce the current and future diabetes burden.

The long-term nature of chronic and progressive diseases like T2D lends itself to the use of simulation modelling. Simulation modelling is increasingly being used to help inform decisions on the allocation of scarce resources and to evaluate the clinical and economic outcomes associated with new public health interventions or clinical treatment strategies [9, 10].

### Key features of T2D modelling

For T2D, there have been numerous modelling studies that have been conducted, investigating both pharmacological and non-pharmacological strategies for disease prevention and management. These studies estimate the costs and benefits across a target population by considering the outcomes of a group of patients (simulation cohort) that is designed to be representative of the target population [11]. In terms of simulation approach, outcomes can be estimated for each individual then averaged across a sufficiently large sample (known as patient-level/microsimulation modelling) or are estimated for a group of individuals without considering the outcomes of individuals in the cohort (known as cohort modelling). Most of the studies make use of Markov-based models using either cohort/microsimulation solution methods, discrete-time microsimulation models or discrete-event microsimulation models to estimate the costs and health benefits of the simulation.

Markov-based models are state-transition models that assess the probabilities of transition to determine if a patient has moved from one state to another at the end of each cycle, with transition to another state relying solely on the current state [12]. Like the Markov-based models, discrete-time microsimulations model disease progression through time using a constant time step and then assess which events have happened at the end of each time step (or cycle). However, the main difference is that each complication is often modelled as a single event and the events are not mutually exclusive. On the other hand, for discrete-event microsimulations, movements between patients’ health states are usually driven by events which may occur at varying times (rather than during cycles of fixed length), and time-to-event distributions are required for each event [13]. Other less common modelling approaches include continuous time models (using differential equations) and object-oriented simulations.

Another key feature of model-based T2D studies is how they assess the robustness of model outputs to input parameter uncertainty (second-order uncertainty). This is often done through deterministic sensitivity analysis, where model inputs are specified as multiple point estimates and varied manually, or through probabilistic sensitivity analysis where rather than being treated as fixed values, model inputs are specified as probability distributions and varied stochastically to provide a probabilistic distribution of model output values. Monte Carlo simulation techniques are often employed to perform probabilistic sensitivity analysis on both cohort or microsimulation models and are also used to account for patient heterogeneity and stochastic uncertainty (first-order uncertainty).

### T2D modelling in LMICs

Most model-based studies on T2D are conducted in HICs and generalised to LMICs, which is not entirely justified given how sub-phenotypes and clinical consequences of T2D vary by ethnic groups (for example, Asians and Africans develop diabetes a decade earlier and at a lower body mass index than those with European ethnicity [14, 15]). While attempts have been made to compile the evidence base and appraise the methodological quality of simulation models and model-based economic evaluations [1, 10, 16–19], no review has been identified that focuses solely on LMICs. We reviewed the use of simulation models and model-based economic evaluations in populations with T2D. The specific objectives were the following:

1. To identify model-based studies on T2D populations investigating both pharmacological and non-pharmacological strategies for T2D management in LMICs.
2. To assess whether all key elements of the modelling procedure were clearly reported according to the Consolidated Health Economic Evaluation Reporting Standards (CHEERS) checklist [20].
3. To summarize and assess the quality and validity of simulation models used in LMICs and discuss knowledge gaps, challenges, and opportunities.

## Materials and methods

Peer-reviewed studies that reported computer simulation models of the natural history of individuals with Type 2 diabetes or used decision models to evaluate the impact of interventions on these populations were identified for this study. Searches were conducted in PubMed (29 June 2020) and followed the Preferred Reporting Items for Systematic Reviews and Meta-Analyses (PRISMA) guidelines [21]. Exact search terms and search strategy are included as supplementary material.

The inclusion/exclusion criteria for study selection are provided in Table 1. Briefly, studies were restricted to those published in the English language since 2000, while only studies from countries classified as low and middle income by the World Bank (2020 classification) were extracted [22]. Studies were excluded from the review if they were clinical, costing or cost-effectiveness/utility studies with no modelling. Given the shorter time frames for within-trial cost effectiveness analyses, these were also excluded. Further, studies where costs and outcomes were calculated for a period less than 5 years were also excluded as most T2D related health problems develop gradually over several years. Additionally, studies focusing on screening for, or preventing T2D rather than post-diagnosis costs and outcomes were excluded.

**Table 1:** Inclusion and exclusion criteria for the review

Parameter	Inclusion criteria	Exclusion criteria
Patient population	Patients with T2DM	Patients with T1D, Gestational diabetes, Healthy individuals, pre-diabetes
Interventions	Modelling studies assessing any pharmacological or non-pharmacological intervention for the treatment of T2D	
Study design	Cost-effectiveness analyses Cost-utility analyses	Within trial Cost-effectiveness analyses, Within trial Cost-utility analyses
	Microsimulations, cohort models	
	Modelling application studies with a time horizon of ≥5 years	Modelling application studies with a time horizon of < 5 years
Country	Low-and-middle income countries	High income countries
Language	Published material in English	Published material not in English
Time frame	Evidence published since 2000 to 30 June 2020	Evidence published prior to 2000

Data on the study setting, intervention type, cost perspective, model type and structures, simulation approach, disease progression, handling of uncertainty, incorporation of treatment effect and outcomes were extracted to give an overview of the model- based studies included in the review. The model structure must have included the progression of T2D (or specific complications) and some health economic outcome, including (but not restricted to) costs, quality-adjusted life-years (QALYs), disability-adjusted life-years (DALYs), life-years (LYs) and incremental cost-effectiveness ratios (ICERs). Studies were then assessed using the Consolidated Health Economic Evaluation Reporting Standards (CHEERS) [20] checklist for economic evaluation of health interventions, which assesses whether all key elements of model structure, parameterisation and approach have been clearly reported.

## Results

### Summary of included studies

**Fig. 1:**
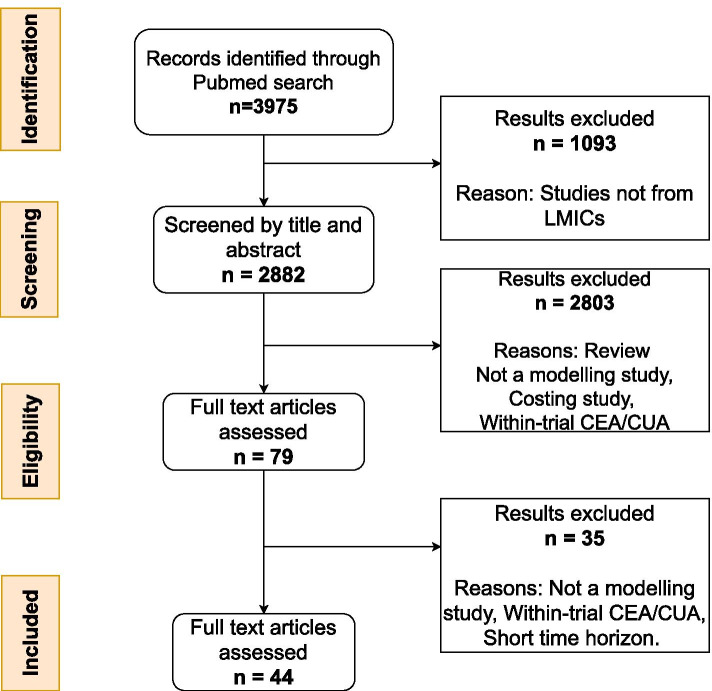
PRISMA diagram showing the flow of publications included and excluded from the review

**Fig. 2:**
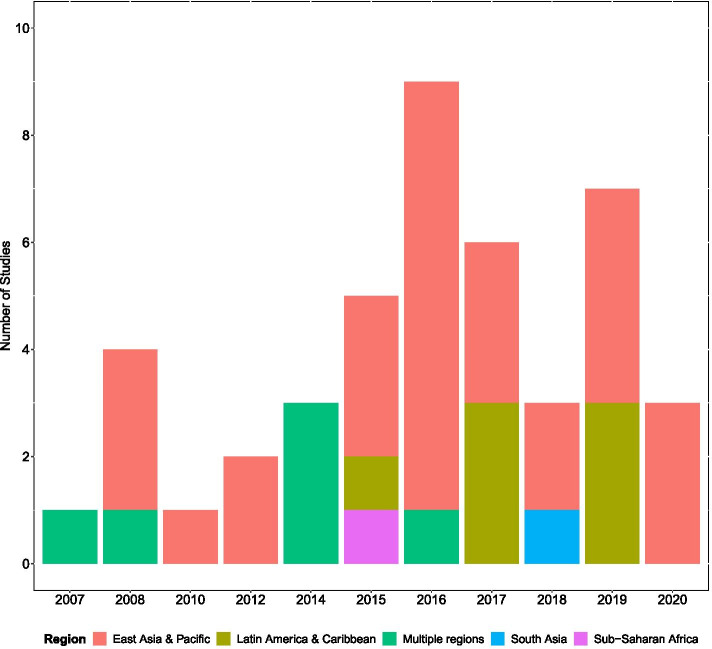
The number of studies in a year by region

The search identified 2882 citations, excluding any duplicate publications and studies from high income countries (Fig. 1). During the screening of titles and abstracts, a further 2803 citations were excluded. Upon full text assessment of the remaining 79 articles, 44 studies met criteria for inclusion. A summary of the study setting, the model structure, simulation approach, complications modelled, the disease progression modelling approach and the handling of uncertainty is provided in Supplementary Table 1. Additional information on the intervention type, cost perspective, treatment effects, model outcomes and data sources is provided in Supplementary Table 2. Of the included studies, 21 (52.3%) were from China while 6(13.6%) were multiple country publications. A majority of the studies (*n* = 29, 65.9%) were exclusively from the East Asia and the Pacific region while 7(15.9%) were from Latin America and the Caribbean (Fig. 2). Only two of the studies, one of which is a multiple country study, included Sub-Saharan African populations (*n* = 2, 4.5%).

All of the 44 studies were cost-effectiveness/utility analyses with 34 (77.3%) evaluating the cost-effectiveness of pharmacological interventions, 4 (9.1%) for obesity surgery and 4 (9.1%) evaluating multiple intervention types. More than half of the studies (*n* = 26, 60.5%) presented results from the healthcare provider perspective, while the rest presented results from the healthcare payer perspective (*n* = 13, 30.2%), and the societal perspective (*n* = 4, 9.1%). In all, 40 studies (90.9%) reported all key elements mentioned in the CHEERS checklist, although only a few studies (*n* = 3, 6.5%) provided the completed checklist as part of the supplementary material.

### Key model characteristics

Of the identified 44 publications, 29(65.9%) of the models were “Markov based” while 15(34.1%) were based on discrete-time microsimulations. In all, 12 (27.3%) of the publications made use of cohort models which estimated the outcomes for the group of patients without explicitly considering the outcomes of each individual patient, while the rest employed patient-level/microsimulation solution approaches.

Most of the studies (*n* = 42, 95.5%) simulated multiple micro-vascular or macro-vascular complications related to type 2 diabetes (Supplementary Table 1), with 33(75.0%) of the studies modelling both micro-vascular and macro-vascular complications. Micro-vascular complications included diabetic nephropathy, neuropathy, and retinopathy while macro-vascular complications included myocardial infarction, coronary artery disease, peripheral arterial disease and stroke. As these complications may take many years to develop, they are often estimated using short-term proxy outcomes, such as glycated haemoglobin levels (HbA1c) [18]. For most of the studies, the impact of the interventions was simulated through changes in risk factors, particularly HbA1c, which was used in 33(75.0%) of the studies. Other risk factors included body mass index (BMI)/weight, hypoglycaemic events, lipid levels and blood pressure. The change in the risk factors subsequently informed the risk of onset of the complications.

Movement between health states depends on the simulation method adopted in the study. In the included studies, 27(61.4%) made use of transition probabilities to simulate disease progression, while 15(34.1%) made use of risk equations, and 2(4.5%) studies used a combination of transition probabilities (for microvascular complications) and risk equations (for macrovascular complications). The risk equations used were the United Kingdom Prospective Diabetes Study (UKPDS) risk equations (*n* = 14, 31.8%) [23–25], and the Risk Equations for Complications Of type 2 Diabetes (RECODe) (*n* = 4, 9.1%) [26]. The majority of the studies assessed the probabilities of moving to another state (Markov models) or the occurrence of events (discrete-time simulations) through 1-year cycles (*n* = 29, 70.7%), and accounted for costs and outcomes over 20 years or more (*n* = 38, 86.4%).

More than half of the studies (n = 29, 65.9%) made use of seven existing validated diabetes models. These validated models included the IQVIA CORE Diabetes Model, formerly known as the IMS CORE Diabetes Model (*n* = 12, 27.3%), Cardiff Diabetes Model (*n* = 7, 15.9%), Chinese Outcomes Model for T2DM (*n* = 4, 9.1%) and the UKPDS Outcomes Model (*n* = 2, 4.5%) [27–30]. All the studies that used existing models provided short descriptive summaries of the model. These models all went through internal validation to ensure the model accurately predicts outcomes from the same trials and datasets from which they were originally developed. Additionally, 6 of the 7 models, corresponding to 28(63.6%) studies, reported external validation which compares a model’s results with that of real-world results that were not used to populate the model (e.g., other clinical trials). This is done in order to ensure predictive validity and accuracy.

### Handling uncertainty

Sensitivity analyses such as the impact of discount rates, time horizons, intervention efficacy and intervention costs were frequently reported. Univariate sensitivity analyses were performed and reported in all but one of the studies included in this review. Most of the studies (*n* = 41, 93.1%) also performed probabilistic sensitivity analysis, through Monte-Carlo simulation though it is not always clear whether first- or second-order uncertainties were considered in the simulations.

### Outcomes and costs

The most reported outcomes were QALYs (*n* = 41, 93.1%) and life years (*n* = 2, 4.5%). All the studies reported direct costs and also ICERs as a summary measure of costs and outcomes. Other reported outcomes were DALYs and cumulative incidence of complications, while 4(9.1%) of the studies accounted for indirect costs.

### Key data sources

Data on the effectiveness of interventions were mainly obtained from literature searches (*n* = 29, 65.9%), and from observational studies (*n* = 9, 20.5%), but most resource-use data and unit costs were obtained through routine data collection from administrative databases and published price lists (*n* = 30, 65.2%). The majority of the studies made use of utility or health related quality of life data obtained from literature reviews (*n* = 35, 87.5%), while baseline patient characteristics of the simulated cohorts including age, sex, duration of diabetes, race/ethnicity, and other modifiable risk factors/biomarkers related to T2D, were often based on RCTs or literature reviews (*n* = 31, 70.5%). The sources of the data used to model the progression of microvascular and/or macrovascular complications varied. The validated models made use of data from RCTs with the most commonly used RCT being the UKPDS and the Diabetes Control and Complications Trial (DCCT) [31–33]. The other studies made use of literature reviews to obtain inputs used to define population characteristics and transition probabilities. There was no mention, however, of whether meta-analysis had been used to pool estimates from the studies that were retrieved from the literature.

## Discussion

Given the high cost and burden of diabetes, there is significant interest in identifying strategies that are cost-effective and delay the onset of T2D related complications. The long-term nature of T2D necessitates the use of simulation modelling in order to extrapolate from short-term empirical studies to predict costs and health benefits over the lifetime of an individual. The current study aimed to critically appraise the use of simulation modelling for long-term T2D management in LMICs. The studies identified were assessed for their technical characteristics while the model-based economic evaluations were additionally assessed for their adherence to reporting standards. Overall, there has been an increase in the number of modelling studies conducted in LMICs, particularly in Asia, with most of the studies being model-based economic evaluations of pharmacological interventions. The paucity of studies exclusively from Africa is somewhat indicative of the focus on communicable disease research in the region [34]. This is worrisome given the expected increase in T2D prevalence and highlights the need for more T2D research in the region. Most of the economic evaluations adhered to reporting standards (CHEERS) which is crucial given the push for more transparent reporting of T2D models and economic evaluations to improve reliability and reproducibility [35]. Despite this, there are key issues that modellers in LMIC populations need to account for.

While most of the studies identified in this review employed state transition (Markov) modelling techniques (Supplementary Table 1), there is no clear consensus on the most appropriate modelling approach [18]. A defining feature of Markov modelling, which is also a limitation, is the ‘memoryless property’ or ‘Markovian assumption’ where future transitions are not dependent on previous states [12]. There are ways of mimicking memory for cohort models such as the introduction of tunnel states, which in turn can lead to state explosion thereby making the model difficult to manage [36, 37]. Willis et al. [37] also identified potentially biased estimates of the incremental cost-effectiveness ratio (ICER) arising from “uncaptured” patient heterogeneity as a key shortcoming of using cohort models in cost-effectiveness analyses. For T2D, this additional complexity is inevitable given the significant heterogeneity existing between patients and the interrelated risks [10].

In contrast with Markov-based cohort models, Markov-based microsimulation models simulate individual patient histories over time, thereby automatically accounting for heterogeneity. Additionally, the Markov assumption is overcome by assigning attributes to individuals that can influence their progression through the model (tracker variables), but which can also be used to update the risk of future events accordingly [38, 39]. A more flexible alternative to Markov modelling identified in this review is discrete-time microsimulation modelling. This approach accounts for an individual’s specific demographic characteristics, risk factors and event history when assigning risks of events/complications occurring and is not restricted to fixed ‘mutually exclusive’ health states as is the case for Markov-based models [40, 41].

There are drawbacks to microsimulation modelling, particularly, the onerous computational and data requirements [10]. In LMICs, national (and ethnic)-specific data with which to develop T2D microsimulation models are seldom available [42]. As a result, most studies investigating the long-term health outcomes and economic consequences of interventions either make use of existing validated diabetes models or use international RCT data to develop the models despite the possible transferability issues [42]. In this study, we identified 7 existing validated models; IMS (IQVIA) CORE Diabetes Model, Cardiff Diabetes model, Chinese Outcomes Model for T2D (COMT), UKPDS Outcomes Model (UKPDS OM), CDC-RTI model, Economic and Health Outcomes (ECHO) model and the Januvia Diabetes Economic (JADE) model [27–30, 43–45]. Of these models, only one was a Markov-based cohort model (CDC-RTI) with the rest being either discrete-time microsimulations (JADE, UKPDS OM, COMT, Cardiff) or Markov-based microsimulations (CORE, ECHO). It is worth noting that of these models, only one was developed in a low- and middle-income country. While many of these models had undergone external validation, it was not always clear, however, whether the models had been validated for the populations of interest.

Previous reviews by Tarride et al., Yi et al., Govan et al., Charokopou et al., and Li et al. have critically appraised the above models, provided comprehensive summaries, and discussed the capabilities and shortcomings of the models [1, 10, 16–19]. They found that the structures of the Markov-based microsimulation models were broadly similar, as were the discrete-time microsimulation models. For the Markov-based microsimulations, each complication was included as a sub-model although the number of health states differed by model. As an example, the nephropathy sub-model in the IMS (IQVIA) CORE Diabetes Model had 7 states while the nephropathy sub-model in the Economic and Health Outcomes (ECHO) model had 4 states. The discrete-time microsimulation models, on the other hand, are based on a series of risk equations. In principle, risk equations predict the annual probability of specific micro/macro-vascular end points based on patient demographics, duration of diabetes, risk factor levels, and history of micro/macro-vascular complications, and then employ Monte Carlo methods to predict the occurrence of long-term complications.

For most of the validated models, data from the UKPDS were used to derive the risk equations or determine transition probabilities that predict the long term T2D complications [1, 16]. Tarride et al. [1] highlight some issues associated with using UKPDS data. First, there is an issue of generalizability to more ethnically diverse populations and different ethnic groups given how 83% of the UKPDS participants had European ethnicity with a median age of 54 years (IQR 48–60 years) [31]. Second, there is a likelihood that treatment regimes evaluated in the study may not correspond to current practice [1, 46]. Further, exposure to risk factors, disease incidence, and standards of care for T2D are also likely to be notably different now compared to when the original study was conducted (1977-1997). This may affect the applicability of most validated T2D models to different LMIC populations. In addition, to use a validated T2D model, it is often assumed that the overall predicted rate of events is applicable to a similar population as the one used for model development or external validation. This, however, is not always the case considering there are notable differences in the proportion of baseline risk factors such as demographic, ethnic, lifestyle, and treatment options in different regions.

Strategies for modifying existing models have been proposed for use in populations with dissimilar characteristics to the ones from which the models were derived, and where the appropriate data and resources to develop new models are not available. The aim is to improve the performance of risk prediction models which may over- or under-estimate risk in different populations [47]. One such strategy is recalibration of the underlying risk equations or transition probabilities using local data on risk factor distributions and the incidence of micro/macro-vascular complications [48]. The strategy assumes that the associations between different risk factors and complications are constant, hence adjusting the equations for different risk factor distributions and different background incidence of micro/macro-vascular complications is adequate [48]. Of the studies that made use of existing validated models, 4 of them used a model, the Chinese Outcomes Model for T2DM (COMT) [29] whose underlying risk equations had been recalibrated using Chinese-specific epidemiological data. Specifically, the Risk Equations for Complications of Type 2 Diabetes (RECODe), which were developed for the United States population, were recalibrated to suit the Chinese population by “adding an adjustment regulator to the original linear predictor within the risk equations” with a view to eliminating the over- or under-estimation of risk in the Chinese population [29, 49].

While recalibration of Markov-based models is not as common, guidance from the International Society for Pharmacoeconomics and Outcomes Research (ISPOR) suggests that adaptation of previously published or validated models for “local” use is possible [50]. According to Mullins et al. [50], key to the adaptation process is the use of country/region specific epidemiologic data to adjust the baseline characteristics of the simulated cohort, possibly generate new transition probabilities and adjust the initial distributions for Markov-based models. Further, local cost, health state preferences and utilities data are also important for the adaptation.

When either developing a new model or adapting/recalibrating an existing one, the availability of reliable and applicable data is key. The scarcity of robust T2D data from LMIC populations continues to be a major hindrance in conducting model-based analyses that focuses on these populations [42, 46]. There are other data requirements for model-based analyses which include baseline patient characteristics of the simulated cohort, clinical effectiveness data, utility data and resource use (cost) data. Two main strategies are employed to get these data: collecting existing data through systematic literature reviews and meta-analyses, or collecting the data in a clinical trial [42]. Other possible sources include observational studies, routine data collection and expert review. Baik et al. [42] advocate for increased use of observational study data on T2D in LMICs despite potential selection bias issues when compared to RCTs. Specifically, they highlight how parameters such as health-related quality of life, and resource use are less likely to be subject to selection bias if derived from observational studies. Considering this, observational studies, which are more readily available in LMICs, can bridge the gaps in data required for economic evaluations and model-based analyses.

### Strengths and limitations

Despite being the first to provide a snapshot of T2D modelling in LMICs and a synthesis of key considerations in the design and development of T2D simulation models in LMICs, this review is not without limitations. First, by extracting only the information related to modelling, valuable insights on the efficacy and cost-effectiveness of the interventions being modelled are missed. A more comprehensive evaluation of these studies in relation to the actual decision problem being addressed and identifying the most cost-effective interventions would be possible in future. Second, the exclusion criteria such as omitting published material not in English or excluding studies on prevention of or screening for T2D may have omitted some studies that would have added to the discourse. Lastly, a more appropriate checklist for input data reporting in T2D model-based analyses is the recently developed Diabetes Modelling Input Checklist [35]. However, considering the significant overlap with the CHEERS checklist and how most studies in our review were published before it was developed, the CHEERS checklist was deemed adequate for the task. Despite these shortcomings, we believe that no major studies that can change the results of this review have been missed.

## Conclusions

In summary, the use of simulation modelling in the management of T2D has been steadily increasing in LMICs, possibly allowing improved decision making on the optimal allocation of scarce resources and the improvement of patient outcomes. Nevertheless, there is an urgent need to invest in evaluating therapeutic and policy interventions related to T2D in LMICs through simulation modelling, especially with local research data. In addition, clinical and observational research on T2D in LMICs is clearly warranted, particularly among sub-Saharan African populations given the paucity of model-based analyses in the region. Finally, it is important to improve transparency and credibility in the reporting of input data underlying model-based economic analyses, and modelling studies, respectively, in LMICs. The use of the Diabetes Modelling Input checklist and the CHEERS checklist is therefore encouraged.

## Supplementary Information

**Additional file 1: Supplementary Table 1**. Summary of model structural framework and the method of disease progression across identified diabetes models. **Supplementary Table 2**. Summary of key methodological features of type 2 diabetes mellitus models. **Supplementary Table 3**. PubMed database search strategy (29 June 2020).

## Data Availability

Data sharing not applicable to this article as no datasets were generated or analysed during the current study.

## Abbreviations

CHEERS: Consolidated Health Economic Evaluation Reporting Standards
DALYs: Disability-adjusted life-years
HIC: High-income country
ICERs: Incremental cost-effectiveness ratios
IDF: International Diabetes Federation
LMIC: Low-middle income country
LYs: Life-years
PRISMA: Preferred Reporting Items for Systematic Reviews and Meta-Analyses
QALYs: Quality-adjusted life-years
RCT: Randomised Controlled Trials
RECODe: Risk Equations for Complications Of type 2 Diabetes
T1D, T2D: Type 1 or Type 2 Diabetes Mellitus
UKPDS: United Kingdom Prospective Diabetes Study
