# Supplementary material for: A review of simulation models for the long-term management of type 2 diabetes in low-and-middle income countries
Source: BMC Health Serv Res. 2021 Dec 6;21:1313. doi: 10.1186/s12913-021-07324-0 (PMC8650231; doi:10.1186/s12913-021-07324-0)
Supplement: Supplementary file 1 — Additional file 1: Supplementary Table 1. Summary of model structural framework and the method of disease progression across identified diabetes models. Supplementary Table 2. Summary of key methodological features of type 2 diabetes mellitus models. Supplementary Table 3. PubMed database search strategy (29 June 2020). [file 12913_2021_7324_MOESM1_ESM.docx]

**Title:** A review of simulation models for the long-term management of Type 2 Diabetes in Low- and-Middle Income countries.

**Authors:** Elton Mukonda1, Susan Cleary2, Maia Lesosky1

**Affiliation:**

1Division of Epidemiology & Biostatistics, School of Public Health & Family Medicine, University of Cape Town, Cape Town, South Africa;

2Health Economics Unit, School of Public Health & Family Medicine, University of Cape Town, Cape Town, South Africa;

# Supplementary material

**Supplementary Table 1 Summary of model structural framework and the method of disease progression across identified diabetes models**

| **Source** | **Setting** | **Model type** | **Complications** | **Simulation**  **Method** | **Disease progression** | **Cycle**  **length** | **Time horizon**  **(years)** | **Monte Carlo**  **Methods** |
| --- | --- | --- | --- | --- | --- | --- | --- | --- |
| Annemans et al,2007 [1] | Multiple countries | Markov model | Diabetic Nephropathy | Cohort | Transition probabilities | 1-year | 25 | Yes |
| Assumpção et  al,2019 [2] | Brazil | Decision tree and Markov model | Fatal and non-fatal myocardial  infarction, and death. | Cohort | Transition  probabilities | 1-year | 10 | Yes |
| Basu et al,2016  [3] | Multiple  countries | Discrete-time Monte Carlo  microsimulation | Microvascular /  Macrovascular | Patient-level | Risk equations | 1-year | Lifetime | Yes |
| Cai et al,2019 [4] | China | Discrete-time Monte Carlo microsimulation (Cardiff Diabetes Model) | Microvascular / Macrovascular | Patient-level | Risk equations | 6-months | 40 | Yes |
| Cheng et al,2019 [5] | China | Discrete-time Monte Carlo microsimulation (Chinese Outcomes  Model for T2DM (COMT)) | Microvascular / Macrovascular | Patient-level | Risk equations | 1-year | Lifetime | Yes |
| Chirakup et al,2008 [6] | Thailand | Markov- based Monte Carlo microsimulation (IMS CORE Diabetes  Model) | Microvascular / Macrovascular | Patient-level | Transition probabilities | 1-year | 40 | Yes |
| Cohen et al,2017 [7] | Brazil | Markov- based Monte Carlo microsimulation | Macrovascular (CVDs) | Patient-level | Transition probabilities | 1-year | 20 | Yes |
| De-Oliveira et  al,2017 [8] | Brazil | Markov model | Microvascular /  Macrovascular | Cohort | Transition  probabilities | 1-year | 10 | Yes |
| Deng et al,2015  [9] | China | Discrete-time Monte Carlo  microsimulation (Cardiff Diabetes Model) | Microvascular /  Macrovascular | Patient-level | Risk equations | 6-months | 40 | Yes |
| Flessa et al,2015 [10] | Cambodia | Markov model | Microvascular / Macrovascular | Cohort | Transition probabilities | 1-year | 20 | No |
| Gao et al,2012  [11] | China | Discrete-time Monte Carlo  microsimulation (UKPDS Outcomes Model) | Microvascular /  Macrovascular | Patient-level | Risk equations | - | 30 | Yes |
| Gil-Rojas et al,2019 [12] | Colombia | Markov model | Macrovascular (CVDs) | Cohort | Transition probabilities | 1-year | 5 | Yes |
| Gilmer et al,2019  [13] | Mexico | Discrete-time Monte Carlo  microsimulation (UKPDS Outcomes Model) | Microvascular /  Macrovascular | Patient-level | Risk equations | - | Lifetime | Yes |
| Gu et al,2015 [14] | China | Discrete-time Monte Carlo microsimulation (Cardiff Diabetes Model) | Microvascular / Macrovascular | Patient-level | Risk equations | 6-months | 40 | Yes |

**Supplementary Table 1 Summary of model structural framework and the method of disease progression across identified studies (cont.)**

| **Source** | **Setting** | **Model types** | **Complications** | **Simulation**  **Method** | **Disease progression** | **Cycle**  **length** | **Time horizon**  **(years)** | **Monte Carlo**  **Methods** |
| --- | --- | --- | --- | --- | --- | --- | --- | --- |
| Gu et al,2016 [15] | China | Discrete-time Monte Carlo microsimulation (Cardiff Diabetes Model) | Microvascular / Macrovascular | Patient-level | Risk equations | 6-months | 40 | Yes |
| Gu et al,2016  [16] | China | Discrete-time Monte Carlo microsimulation  (Cardiff Diabetes Model) | Microvascular /  Macrovascular | Patient-level | Risk equations | 6-months | 40 | Yes |
| Gu et al,2017 [17] | China | Discrete-time Monte Carlo microsimulation (Cardiff Diabetes Model) | Microvascular / Macrovascular | Patient-level | Risk equations | 6-months | 40 | Yes |
| Gupta et al,2014  [18] | Multiple  countries | Markov- based Monte Carlo microsimulation  (IMS CORE Diabetes Model) | Microvascular /  Macrovascular | Patient-level | Transition probabilities | 1-year | 30 | Yes |
| Gupta et al,2018 [19] | India | Markov- based Monte Carlo microsimulation (Economic and Health Outcomes (ECHO)-T2DM) | Microvascular / Macrovascular | Patient-level | Transition probabilities/ Risk equations | 1-year | 20 | Yes |
| Home et al,2014  [20] | Multiple  countries | Markov- based Monte Carlo microsimulation  (IMS CORE Diabetes Model) | Microvascular /  Macrovascular | Patient-level | Transition probabilities | 1-year | 30 | Yes |
| Hou et al,2019  [21] | China | Discrete-time Monte Carlo microsimulation  (Chinese Outcomes Model for T2DM (COMT)) | Microvascular /  Macrovascular | Patient-level | Risk equations | 1-year | Lifetime | Yes |
| Jiang et al,2019 [22] | China | Markov model | Macrovascular (CVDs) | Cohort | Transition probabilities | 1-year | 40 | Yes |
| Lasalvia et  al,2017 [23] | Colombia | Markov model | Microvascular | Cohort | Transition probabilities | 6-months | 5 | Yes |
| Li et al,2018 [24] | China | Discrete-time Monte Carlo microsimulation (Chinese Outcomes Model for T2DM (COMT)) | Microvascular / Macrovascular | Patient-level | Risk equations | 1-year | Lifetime | Yes |
| Mash et al,2015  [25] | South  Africa | Markov micro-simulation | Macrovascular | Patient-level | Transition probabilities | - | 10,30, Lifetime | Yes |
| Neslusan et al,2015 [26] | Mexico | Markov- based Monte Carlo microsimulation (Economic and Health Outcomes (ECHO)-T2DM) | Microvascular / Macrovascular | Patient-level | Transition probabilities/ Risk equations | 1-year | 20 | Yes |
| Nian et al,2020  [27] | China | Discrete-time Monte Carlo microsimulation  (Chinese Outcomes Model for T2DM (COMT)) | Microvascular /  Macrovascular | Patient-level | Risk equations | 1-year | Lifetime | Yes |
| Ou et al,2016 [28] | China | Markov model (CDC-RTI Diabetes Cost- effectiveness Model) | Macrovascular (CVDs) | Cohort | Transition probabilities | 1-year | 35 | Yes |
| Palmer et  al,2008 [29] | China | Markov- based Monte Carlo microsimulation  (IMS CORE Diabetes Model) | Microvascular /  Macrovascular | Patient-level | Transition probabilities | 1-year | 30 | Yes |

**Supplementary Table 1 Summary of model structural framework and the method of disease progression across identified diabetes models (cont.)**

| **Source** | **Setting** | **Model type** | **Complications** | **Simulation**  **Method** | **Disease progression** | **Cycle**  **length** | **Time horizon**  **(years)** | **Monte Carlo**  **Methods** |
| --- | --- | --- | --- | --- | --- | --- | --- | --- |
| Palmer et al,2010 [30] | China | Markov- based Monte Carlo microsimulation (IMS CORE Diabetes Model) | Microvascular / Macrovascular | Patient-level | Transition probabilities | 1-year | 30 | Yes |
| Permsuwan et  al,2016 [31] | Thailand | Markov- based Monte Carlo microsimulation  (IMS CORE Diabetes Model) | Microvascular /  Macrovascular | Patient-level | Transition probabilities | 1-year | Lifetime | Yes |
| Permsuwan et al,2016 [32] | Thailand | Markov- based Monte Carlo microsimulation (IMS CORE Diabetes Model) | Microvascular / Macrovascular | Patient-level | Transition probabilities | 1-year | Lifetime | Yes |
| Permsuwan et  al,2016 [33] | Thailand | Markov- based Monte Carlo microsimulation  (IMS CORE Diabetes Model) | Microvascular /  Macrovascular | Patient-level | Transition probabilities | 1-year | Lifetime | Yes |
| Permsuwan et al,2017 [34] | Thailand | Markov- based Monte Carlo microsimulation (IMS CORE Diabetes Model) | Microvascular / Macrovascular | Patient-level | Transition probabilities | 1-year | 50 | Yes |
| Schwarz et  al,2008 [35] | Multiple  countries | Discrete-time Monte Carlo microsimulation  (Januvia Diabetes Economic (JADE) Model) | Microvascular /  Macrovascular | Patient-level | Risk equations | - | Lifetime | Yes |
| Shafie et al,2014  [36] | Multiple  countries | Markov- based Monte Carlo microsimulation  (IMS CORE Diabetes Model) | Microvascular /  Macrovascular | Patient-level | Transition probabilities | 1-year | 30 | Yes |
| Shao et al,2016 [37] | China | Discrete-time Monte Carlo microsimulation (Cardiff Diabetes Model) | Microvascular / Macrovascular | Patient-level | Risk equations | 6-months | 40 | Yes |
| Wan et al,2020  [38] | China | Markov model | Not Specified | Cohort | Transition probabilities | 1-year | 40 | Yes |
| Wang et al,2020 [39] | China | Markov model | Not Specified | Cohort | Transition probabilities | 3-months | 5 | No |
| Wu et al,2017 [40] | China | Markov model | Diabetic kidney disease (DKD)/ Diabetic  nephropathy | Cohort | Transition probabilities | 1-year | Lifetime | Yes |
| Wu et al,2018  [41] | China | Markov model | Diabetic Foot Ulcer and  Amputation | Cohort | Transition probabilities | 1-month | Lifetime | Yes |
| Xie et al,2008 [42] | China | Markov- based Monte Carlo microsimulation | Microvascular / Macrovascular | Patient-level | Transition probabilities | 1-year | 11 | Yes |
| Yang et al,2012  [43] | China | Markov- based Monte Carlo microsimulation  (IMS CORE Diabetes Model) | Microvascular /  Macrovascular | Patient-level | Transition probabilities | 1-year | 30 | Yes |
| Zhang et al,2016 [44] | China | Markov- based Monte Carlo microsimulation (IMS CORE Diabetes Model) | Microvascular / Macrovascular | Patient-level | Transition probabilities | 1-year | 30 | Yes |

**Supplementary Table 2 Summary of key methodological features of type 2 diabetes mellitus models**

| **Source** | **Intervention type** | **Perspective** | **Treatment effects** | **Outcome measures** | **Discount** | **Effectiveness data** | **Cost data** | **Utility data** | **Parameter data** | **Internal / External validation** |
| --- | --- | --- | --- | --- | --- | --- | --- | --- | --- | --- |
| Annemans et  al,2007 [1] | Pharmacological/  Screening | Provider | Blood pressure | QALYs/Costs/ICER | 3% | RCT | Routine data  collection | - | RCT | No |
| Assumpção et al,2019 [2] | Obesity Surgery | Provider | BMI/body weight | QALYs/Costs/ICER | 5% | Literature review | observational study | observational study | Literature review | No |
| Basu et al,2016  [3] | Pharmacological/  Policy | Societal | HbA1c, blood pressure,  lipid levels | DALYs/Costs/ICER | 3% | Literature  review | Routine data  collection | Literature  review | Literature  review | Yes |
| Cai et al,2019 [4] | Pharmacological | Payer | HbA1c, BMI/body weight, hypoglycaemic  events | QALYs/Costs/ICER | 3% | Literature review | Routine data collection | Literature review | Literature review | Yes |
| Cheng et al,2019 [5] | Pharmacological | Provider | HbA1c, hypoglycaemic events | QALYs/Costs/ICER | 5% | Literature review | Literature review/Routine  data collection | Literature review | Literature review | Yes |
| Chirakup et al,2008 [6] | Pharmacological | Provider | HbA1c, lipid levels | QALYs/Costs/ICER | 3% | Literature review | Routine data collection | Literature review | Literature review | Yes |
| Cohen et  al,2017 [7] | Obesity Surgery | Provider | BMI/body weight | QALYs/Costs/ICER | 5% | Literature  review | Routine data  collection | Literature  review | Literature  review | Yes |
| De-Oliveira et al,2017 [8] | Pharmacological | Provider | HbA1c, blood pressure, BMI/body weight, lipid levels, hypoglycaemic  events | LYs/Costs/ICER | 5% | Literature review | Routine data collection | - | Literature review | No |
| Deng et al,2015 [9] | Pharmacological | Societal | HbA1c, blood pressure, BMI/body weight, lipid levels, hypoglycaemic  events | QALYs/Costs/ICER | 3% | Literature review | Routine data collection | Literature review | Literature review | Yes |
| Flessa et  al,2015 [10] | Pharmacological | Provider | - | LYs/Costs/ICER | 5% | Literature  review | Literature  review | - | Literature  review | No |
| Gao et al,2012 [11] | Pharmacological | Provider | HbA1c, blood pressure, body weight, lipid levels | QALYs/Costs/ICER | 3% | RCT/Literature review | routine data collection | Literature review | RCT | Yes |
| Gil-Rojas et  al,2019 [12] | Obesity Surgery | Provider | blood pressure,  BMI/body weight | QALYs/Costs/ICER | 5% | Literature  review | routine data  collection | Literature  review | Literature  review | No |
| Gilmer et al,2019 [13] | Policy | Provider | HbA1c, blood pressure, lipid levels | QALYs/Costs/ICER | 3% | RCT | Literature review | Literature review | RCT | Yes |

**Supplementary Table 2 Summary of key methodological features of type 2 diabetes mellitus models(cont..)**

| **Source** | **Intervention type** | **Perspective** | **Treatment effects** | **Outcome measures** | **Discount** | **Effectiveness data** | **Cost data** | **Utility data** | **Paramete r data** | **Internal / External validation** |
| --- | --- | --- | --- | --- | --- | --- | --- | --- | --- | --- |
| Gu et al,2015  [14] | Pharmacological | Payer | HbA1c, BMI/body weight,  hypoglycaemic events | QALYs/Costs/ICER | 3% | Literature  review | Routine data  collection | Literature  review | Literature  review | Yes |
| Gu et al,2016  [15] | Pharmacological | Payer | HbA1c, BMI/body weight,  hypoglycaemic events | QALYs/Costs/ICER | 3% | Literature  review | Routine data  collection | Literature  review | Literature  review | Yes |
| Gu et al,2016 [16] | Pharmacological | Payer | HbA1c, BMI/body weight, hypoglycaemic events | QALYs/Costs/ICER | 3% | Literature review | Routine data collection | Literature review | Literature review | Yes |
| Gu et al,2017 [17] | Pharmacological | Payer | HbA1c, blood pressure, BMI/body weight, lipid levels,  hypoglycaemic events | QALYs/Costs/ICER | 3% | Literature review | Routine data collection | Literature review | Literature review | Yes |
| Gupta et  al,2014 [18] | Pharmacological | Provider | HbA1c | QALYs/Costs/ICER | 3% | observational  study | observational  study | observational  study | Literature  review | Yes |
| Gupta et al,2018 [19] | Pharmacological | Provider | HbA1c, blood pressure, BMI/body weight | QALYs/Costs/ICER | 5% | Literature review |  | Literature review | Literature review | Yes |
| Home et  al,2014 [20] | Pharmacological | Provider | HbA1c, BMI/body weight,  hypoglycaemic events | QALYs/Costs/ICER | 3% | observational  study | observational  study | observational  study | Literature  review | Yes |
| Hou et al,2019 [21] | Pharmacological | Provider | HbA1c, blood pressure, BMI/body weight | QALYs/Costs/ICER | 5% | Literature review | Literature review/ Routine data  collection | Literature review | Literature review | Yes |
| Jiang et al,2019 [22] | Pharmacological | Provider | CVD reductions | QALYs/Costs/ICER | 3% | Literature review | Routine data collection | Literature review | Literature review | No |
| Lasalvia et al,2017 [23] | Pharmacological | Provider | HbA1c, blood pressure, BMI/body weight, lipid levels,  hypoglycaemic events | QALYs/Costs/ICER | 5% | Literature review | Routine data collection | Literature review | Literature review | No |
| Li et al,2018 [24] | Pharmacological | Provider | lipid levels | QALYs/Costs/ICER | 5% | Literature review | Literature review/ Routine data  collection | Literature review | Literature review | Yes |
| Mash et  al,2015[25] | Policy | Societal | Blood pressure, BMI/body  weight, lipid levels | QALYs/Costs/ICER |  | RCT | RCT | - | - | No |
| Neslusan et al,2015 [26] | Pharmacological | Provider | HbA1c, blood pressure, BMI/body weight, lipid levels,  hypoglycaemic events | QALYs/Costs/ICER | 5% | RCT/Literature review | Routine data collection | Literature review | Literature review | Yes |

| Nian et al,2020 [27] | Pharmacological | Provider | HbA1c, blood pressure, BMI/body weight | QALYs/Costs/ICER | 5% | Literature review | Literature review/ Routine data  collection | Literature review | Literature review | Yes |
| --- | --- | --- | --- | --- | --- | --- | --- | --- | --- | --- |

**Supplementary Table 2 Summary of key methodological features of type 2 diabetes mellitus models(cont..)**

| **Source** | **Intervention type** | **Perspective** | **Treatment effects** | **Outcome measures** | **Discount** | **Effectiveness data** | **Cost data** | **Utility data** | **Parameter data** | **Internal / External**  **validation** |
| --- | --- | --- | --- | --- | --- | --- | --- | --- | --- | --- |
| Ou et al,2016  [28] | Pharmacological | Payer | HbA1c | QALYs/Costs/ICER | 3% | observational  study | Routine data  collection | - | Literature  review | Yes |
| Palmer et al,2008 [29] | Pharmacological | Payer | HbA1c, BMI/body weight, hypoglycaemic events | QALYs/Costs/ICER | 3% | observational study | Routine data collection | Literature review | Literature review | Yes |
| Palmer et  al,2010 [30] | Pharmacological | Payer | HbA1c, BMI/body weight | QALYs/Costs/ICER | 3% | RCT | Routine data  collection | Literature  review | Literature  review | Yes |
| Permsuwan et al,2016 [31] | Pharmacological | Provider | HbA1c, BMI/body weight, hypoglycaemic events | QALYs/Costs/ICER | 3% | Literature review | Routine data collection | Literature review | Literature review | Yes |
| Permsuwan et  al,2016 [32] | Pharmacological | Provider | HbA1c, BMI/body weight,  hypoglycaemic events | QALYs/Costs/ICER | 3% | Literature  review | Routine data  collection | Literature  review | Literature  review | Yes |
| Permsuwan et al,2016 [33] | Pharmacological | Provider | HbA1c, hypoglycaemic events | QALYs/Costs/ICER | 3% | observational study | Routine data collection | Literature review | Literature review | Yes |
| Permsuwan et  al,2017 [34] | Pharmacological | Payer | HbA1c, BMI/body weight,  hypoglycaemic events | QALYs/Costs/ICER | 3% | Literature  review | Routine data  collection | Literature  review | Literature  review | Yes |
| Schwarz et al,2008 [35] | Pharmacological |  | HbA1c, BMI/body weight, hypoglycaemic events | QALYs/Costs/ICER | 3% | Literature review | Routine data collection | Literature review | Literature review | Yes |
| Shafie et  al,2014 [36] | Pharmacological | Provider | HbA1c | QALYs/Costs/ICER | 3% | observational  study | observational  study | observational  study | Literature  review | Yes |
| Shao et al,2016  [37] | Pharmacological | Payer | HbA1c, BMI/body weight,  hypoglycaemic events | QALYs/Costs/ICER | 3% | Literature  review | Routine data  collection | Literature  review | Literature  review | Yes |
| Wan et al,2020 [38] | Obesity Surgery | Payer | blood pressure, BMI/body weight | QALYs/Costs/ICER | 3% | Literature review | Routine data collection | Literature review | Literature review | No |
| Wang et al,2020 [39] | Lifestyle, Pharmacological,  Policy | Payer | HbA1c | QALYs/Costs/ICER | 3% | observational study | Routine data collection | Literature review | observational study | No |
| Wu et al,2017  [40] | Pharmacological | Provider | Blood pressure | QALYs/Costs/ICER | 5% | Literature  review | Literature  review | Literature  review | Literature  review | No |
| Wu et al,2018 [41] | Pharmacological, Policy | Provider | BMI/body weight | QALYs/Costs/ICER | 5% | Literature review | Literature review | Literature review | Literature review | No |
| Xie et al,2008  [42] | Pharmacological | Provider | HbA1c, FPG | QALYs/Costs/ICER | 3% | Literature  review | Literature  review | Literature  review | Literature  review | No |
| Yang et al,2012 [43] | Pharmacological | Payer | HbA1c, BMI/body weight, hypoglycaemic events | QALYs/Costs/ICER | 3% | observational study | Routine data collection | Literature review | Literature review | Yes |

| Zhang et al,2016 [44] | Pharmacological | Societal | HbA1c, blood pressure, BMI/body weight, lipid levels,  hypoglycaemic events | QALYs/Costs/ICER | 3% | observational study | Routine data collection | Literature review | Literature review | Yes |
| --- | --- | --- | --- | --- | --- | --- | --- | --- | --- | --- |

**Supplementary Table 3 PubMed database search strategy ( 29 June 2020)**

| **Search number** | **Query** | **Filters** | **Results** |
| --- | --- | --- | --- |
| 1 | diabet* OR diabetes OR NIDDM OR IDDM OR DM OR "insulin resistance" OR  diabetolog* |  | 840,105 |
| 2 | Diabetes mellitus [MeSH Terms] |  | 423,032 |
| 3 | 1 OR 2 |  | 840,105 |
| 4 | economic evaluation OR economic- evaluation |  | 108,396 |
| 5 | simulation model OR simulation  modelling |  | 376,255 |
| 6 | microsimulation |  | 1,350 |
| 7 | markov model OR markov modelling |  | 23,247 |
| 8 | cost utility OR cost-utility OR CUA |  | 244,015 |
| 9 | cost-effectiveness OR cost effectiveness OR CEA |  | 169,363 |
| 10 | cost of illness OR cost-of-illness |  | 40,001 |
| 11 | 4 OR 5 OR 6 OR 7 OR 8 OR 9 OR 10 |  | 782,866 |
| 12 | 3 AND 11 |  | 57,837 |
| 13 | 3 AND 11 | LMIC | 4,980 |
| 14 | 3 AND 11 | LMIC, from 2000 - 2020 | 4,659 |
| 15 | 3 AND 11 | LMIC, English, from 2000 - 2020 | 4,379 |
| 16 | 3 AND 11 | LMIC, English, Humans, from 2000  - 2020 | 3,975 |

# References

1. Annemans L, Demarteau N, Hu S, et al. An Asian regional analysis of cost- effectiveness of early irbesartan treatment versus conventional antihypertensive, late amlodipine, and late irbesartan treatments in patients with type 2 diabetes, hypertension, and nephropathy. Value Health. 2008;11(3):354-364. doi:10.1111/j.1524-4733.2007.00250.x
2. Assumpção RP, Bahia LR, da Rosa MQM, et al. Cost-Utility of Gastric Bypass Surgery Compared to Clinical Treatment for Severely Obese With and Without Diabetes in the Perspective of the Brazilian Public Health System. Obes Surg. 2019;29(10):3202- 3211. doi:10.1007/s11695-019-03957-7
3. Basu S, Shankar V, Yudkin JS. Comparative effectiveness and cost-effectiveness of treat-to-target versus benefit-based tailored treatment of type 2 diabetes in low- income and middle-income countries: a modelling analysis. Lancet Diabetes Endocrinol. 2016;4(11):922-932. doi:10.1016/S2213-8587(16)30270-4
4. Cai X, Shi L, Yang W, et al. Cost-effectiveness analysis of dapagliflozin treatment versus metformin treatment in Chinese population with type 2 diabetes. J Med Econ. 2019;22(4):336-343. doi:10.1080/13696998.2019.1570220
5. Cheng H, Wan X, Ma J, Wu B. Cost-effectiveness of Insulin Degludec Versus Insulin Glargine in Insulin-naive Chinese Patients With Type 2 Diabetes. Clin Ther. 2019;41(3):445-455.e4. doi:10.1016/j.clinthera.2019.01.003
6. Chirakup S, Chaiyakunapruk N, Chaikledkeaw U, et al. Cost-effectiveness analysis of thiazolidinediones in uncontrolled type 2 diabetic patients receiving sulfonylureas and metformin in Thailand. Value Health. 2008;11 Suppl 1:S43-S51. doi:10.1111/j.1524-4733.2008.00366.x
7. Cohen RV, Luque A, Junqueira S, Ribeiro RA, Le Roux CW. What is the impact on the healthcare system if access to bariatric surgery is delayed?. Surg Obes Relat Dis. 2017;13(9):1619-1627. doi:10.1016/j.soard.2017.03.025
8. De Oliveira GL, Guerra Júnior AA, Godman B, Acurcio FA. Cost-effectiveness of vildagliptin for people with type 2 diabetes mellitus in Brazil; findings and implications. Expert Rev Pharmacoecon Outcomes Res. 2017;17(2):109-119. doi:10.1080/14737167.2017.1292852
9. Deng J, Gu S, Shao H, Dong H, Zou D, Shi L. Cost-effectiveness analysis of exenatide twice daily (BID) vs insulin glargine once daily (QD) as add-on therapy in Chinese patients with Type 2 diabetes mellitus inadequately controlled by oral therapies. J Med Econ. 2015;18(11):974-989. doi:10.3111/13696998.2015.1067622
10. Flessa S, Zembok A. Costing of diabetes mellitus type II in Cambodia. Health Econ Rev. 2014;4(1):24. doi:10.1186/s13561-014-0024-4
11. Gao L, Zhao FL, Li SC. Cost-utility analysis of liraglutide versus glimepiride as add-on to metformin in type 2 diabetes patients in China. Int J Technol Assess Health Care. 2012;28(4):436-444. doi:10.1017/S0266462312000608
12. Gil-Rojas Y, Garzón A, Lasalvia P, Hernández F, Castañeda-Cardona C, Rosselli D. Cost-Effectiveness of Bariatric Surgery Compared With Nonsurgical Treatment in People With Obesity and Comorbidity in Colombia. Value Health Reg Issues. 2019;20:79-85. doi:10.1016/j.vhri.2019.01.010
13. Gilmer T, Burgos JL, Anzaldo-Campos MC, Vargas-Ojeda A. Cost-Effectiveness of a Technology-Enhanced Diabetes Care Management Program in Mexico. Value Health Reg Issues. 2019;20:41-46. doi:10.1016/j.vhri.2018.12.006
14. Gu S, Deng J, Shi L, Mu Y, Dong H. Cost-effectiveness of saxagliptin vs glimepiride as a second-line therapy added to metformin in Type 2 diabetes in China. J Med Econ. 2015;18(10):808-820. doi:10.3111/13696998.2015.1049542
15. Gu S, Zeng Y, Yu D, Hu X, Dong H. Cost-Effectiveness of Saxagliptin versus Acarbose as Second-Line Therapy in Type 2 Diabetes in China. PLoS One. 2016;11(11):e0167190. Published 2016 Nov 22. doi:10.1371/journal.pone.0167190
16. Gu S, Mu Y, Zhai S, Zeng Y, Zhen X, Dong H. Cost-Effectiveness of Dapagliflozin versus Acarbose as a Monotherapy in Type 2 Diabetes in China. PLoS One. 2016;11(11):e0165629. Published 2016 Nov 2. doi:10.1371/journal.pone.0165629
17. Gu S, Wang X, Qiao Q, Gao W, Wang J, Dong H. Cost-effectiveness of exenatide twice daily vs insulin glargine as add-on therapy to oral antidiabetic agents in patients with type 2 diabetes in China. Diabetes Obes Metab. 2017;19(12):1688-1697. doi:10.1111/dom.12991
18. Gupta V, Baabbad R, Hammerby E, Nikolajsen A, Shafie AA. An analysis of the cost- effectiveness of switching from biphasic human insulin 30, insulin glargine, or neutral protamine Hagedorn to biphasic insulin aspart 30 in people with type 2 diabetes. J Med Econ. 2015;18(4):263-272. doi:10.3111/13696998.2014.991791
19. Gupta V, Willis M, Johansen P, et al. Long-Term Clinical Benefits of Canagliflozin 100 mg Versus Sulfonylurea in Patients With Type 2 Diabetes Mellitus Inadequately Controlled With Metformin in India. Value Health Reg Issues. 2019;18:65-73. doi:10.1016/j.vhri.2018.06.002
20. Home P, Baik SH, Gálvez GG, Malek R, Nikolajsen A. An analysis of the cost- effectiveness of starting insulin detemir in insulin-naïve people with type 2 diabetes. J Med Econ. 2015;18(3):230-240. doi:10.3111/13696998.2014.985788
21. Hou X, Wan X, Wu B. Cost-Effectiveness of Canagliflozin Versus Dapagliflozin Added to Metformin in Patients With Type 2 Diabetes in China. Front Pharmacol. 2019;10:480. Published 2019 May 8. doi:10.3389/fphar.2019.00480
22. Jiang M, Li P, You JH, et al. Cost-effectiveness analysis of aspirin for primary prevention of cardiovascular events among patients with type 2 diabetes in China. PLoS One. 2019;14(12):e0224580. Published 2019 Dec 2. doi:10.1371/journal.pone.0224580
23. Lasalvia P, Baquero L, Otálora-Esteban M, Castañeda-Cardona C, Rosselli D. Cost Effectiveness of Dulaglutide Compared with Liraglutide and Glargine in Type 2 Diabetes Mellitus Patients in Colombia. Value Health Reg Issues. 2017;14:35-40. doi:10.1016/j.vhri.2016.10.006
24. Li T, Wan X, Ma J, Wu B. Cost-Effectiveness of Primary Prevention with Statin Treatment for Chinese Patients with Type 2 Diabetes. Adv Ther. 2018;35(12):2214- 2223. doi:10.1007/s12325-018-0823-9
25. Mash R, Kroukamp R, Gaziano T, Levitt N. Cost-effectiveness of a diabetes group education program delivered by health promoters with a guiding style in underserved communities in Cape Town, South Africa. *Patient Educ Couns*. 2015;98(5):622-626. doi:10.1016/j.pec.2015.01.005
26. Neslusan C, Teschemaker A, Johansen P, Willis M, Valencia-Mendoza A, Puig A. Cost- Effectiveness of Canagliflozin versus Sitagliptin as Add-on to Metformin in Patients with Type 2 Diabetes Mellitus in Mexico. Value Health Reg Issues. 2015;8:8-19. doi:10.1016/j.vhri.2015.01.002
27. Nian H, Wan X, Ma J, Jie F, Wu B. Economic evaluation of dapagliflozin versus metformin in Chinese patients whose diabetes is inadequately controlled with diet and exercise. Cost Eff Resour Alloc. 2020;18:12. Published 2020 Feb 28. doi:10.1186/s12962-020-00208-w
28. Ou HT, Chen YT, Liu YM, Wu JS. Comparative cost-effectiveness of metformin-based dual therapies associated with risk of cardiovascular diseases among Chinese patients with type 2 diabetes: Evidence from a population-based national cohort in Taiwan. Diabetes Res Clin Pract. 2016;116:14-25. doi:10.1016/j.diabres.2016.03.013
29. Palmer JL, Gibbs M, Scheijbeler HW, et al. Cost-effectiveness of switching to biphasic insulin aspart in poorly-controlled type 2 diabetes patients in China. Adv Ther. 2008;25(8):752-774. doi:10.1007/s12325-008-0080-4
30. Palmer JL, Beaudet A, White J, Plun-Favreau J, Smith-Palmer J. Cost-effectiveness of biphasic insulin aspart versus insulin glargine in patients with type 2 diabetes in China. Adv Ther. 2010;27(11):814-827. doi:10.1007/s12325-010-0078-6
31. Permsuwan U, Dilokthornsakul P, Saokaew S, Thavorn K, Chaiyakunapruk N. Cost- effectiveness of dipeptidyl peptidase-4 inhibitor monotherapy in elderly type 2 diabetes patients in Thailand. Clinicoecon Outcomes Res. 2016;8:521-529. Published 2016 Sep 21. doi:10.2147/CEOR.S113559
32. Permsuwan U, Dilokthornsakul P, Thavorn K, Saokaew S, Chaiyakunapruk N. Cost- effectiveness of dipeptidyl peptidase-4 inhibitor monotherapy versus sulfonylurea monotherapy for people with type 2 diabetes and chronic kidney disease in Thailand. J Med Econ. 2017;20(2):171-181. doi:10.1080/13696998.2016.1238386
33. Permsuwan U, Chaiyakunapruk N, Dilokthornsakul P, Thavorn K, Saokaew S. Long- Term Cost-Effectiveness of Insulin Glargine Versus Neutral Protamine Hagedorn Insulin for Type 2 Diabetes in Thailand. Appl Health Econ Health Policy. 2016;14(3):281-292. doi:10.1007/s40258-016-0228-3
34. Permsuwan U, Thavorn K, Dilokthornsakul P, Saokaew S, Chaiyakunapruk N. Cost- effectiveness of insulin detemir versus insulin glargine for Thai type 2 diabetes from a payer's perspective. J Med Econ. 2017;20(9):991-999. doi:10.1080/13696998.2017.1347792
35. Schwarz B, Gouveia M, Chen J, et al. Cost-effectiveness of sitagliptin-based treatment regimens in European patients with type 2 diabetes and haemoglobin A1c above target on metformin monotherapy. Diabetes Obes Metab. 2008;10 Suppl 1:43-55. doi:10.1111/j.1463-1326.2008.00886.x
36. Shafie AA, Gupta V, Baabbad R, Hammerby E, Home P. An analysis of the short- and long-term cost-effectiveness of starting biphasic insulin aspart 30 in insulin-naïve people with poorly controlled type 2 diabetes. Diabetes Res Clin Pract. 2014;106(2):319-327. doi:10.1016/j.diabres.2014.08.024
37. Shao H, Zhai S, Zou D, et al. Cost-effectiveness analysis of dapagliflozin versus glimepiride as monotherapy in a Chinese population with type 2 diabetes mellitus. Curr Med Res Opin. 2017;33(2):359-369. doi:10.1080/03007995.2016.1257978
38. Wan B, Fang N, Guan W, et al. Cost-Effectiveness of Bariatric Surgery versus Medication Therapy for Obese Patients with Type 2 Diabetes in China: A Markov Analysis. J Diabetes Res. 2019;2019:1341963. Published 2019 Dec 19. doi:10.1155/2019/1341963
39. Wang H, Wang M, Wang J, et al. Cost-effectiveness analysis of comprehensive intervention programs to control blood glucose in overweight and obese type 2 diabetes mellitus patients based on a real-world setting: Markov modeling. Ann Transl Med. 2019;7(22):676. doi:10.21037/atm.2019.10.38
40. Wu B, Zhang S, Lin H, Mou S. Prevention of renal failure in Chinese patients with newly diagnosed type 2 diabetes: A cost-effectiveness analysis. J Diabetes Investig. 2018;9(1):152-161. doi:10.1111/jdi.12653
41. Wu B, Wan X, Ma J. Cost-effectiveness of prevention and management of diabetic foot ulcer and amputation in a health resource-limited setting. J Diabetes. 2018;10(4):320-327. doi:10.1111/1753-0407.12612
42. Xie X, Vondeling H. Cost-utility analysis of intensive blood glucose control with metformin versus usual care in overweight type 2 diabetes mellitus patients in Beijing, P.R. China. Value Health. 2008;11 Suppl 1:S23-S32. doi:10.1111/j.1524- 4733.2008.00363.x
43. Yang L, Christensen T, Sun F, Chang J. Cost-effectiveness of switching patients with type 2 diabetes from insulin glargine to insulin detemir in Chinese setting: a health economic model based on the PREDICTIVE study. Value Health. 2012;15(1 Suppl):S56-S59. doi:10.1016/j.jval.2011.11.018
44. Zhang X, Liu S, Li Y, Wang Y, Tian M, Liu G. Long-Term Effectiveness and Cost- Effectiveness of Metformin Combined with Liraglutide or Exenatide for Type 2 Diabetes Mellitus Based on the CORE Diabetes Model Study. PLoS One. 2016;11(6):e0156393. Published 2016 Jun 15. doi:10.1371/journal.pone.0156393.
